# An overview on ELISA techniques for FMD

**DOI:** 10.1186/1743-422X-8-419

**Published:** 2011-09-04

**Authors:** Li-na Ma, Jie Zhang, Hao-tai Chen, Jian-hua Zhou, Yao-zhong Ding, Yong-sheng Liu

**Affiliations:** 1 State Key Laboratory of Veterinary Etiological Biology, National Foot and Mouth Disease Reference Laboratory, Lanzhou Veterinary Research Institute, Chinese Academy of Agricultural Sciences, Lanzhou 730046, China

## Abstract

**Background:**

FMD is one of the major causes of economic loss of cloven-hoofed animals in the world today. The assessment of dominant genotype/lineage and prevalent trends and confirmation the presence of infection or vaccination not only provides scientific basis and first-hand information for appropriate control measure but also for disease eradication and regaining FMD free status following an outbreak. Although different biological and serological approaches are still applied to study this disease, ELISA test based on the distinct format, antigen type and specific antibody reinforce its predominance in different research areas of FMD, and this may replace the traditional methods in the near future. This review gives comprehensive insight on ELISA currently available for typing, antigenic analysis, vaccination status differentiation and surveillance vaccine purity and content at all stages of manufacture in FMDV. Besides, some viewpoint about the recent advances and trends of ELISA reagent for FMD are described here.

**Methods:**

More than 100 studies regarding ELISA method available for FMD diagnosis, antigenic analysis and monitor were thoroughly reviewed. We investigated previous sagacious results of these tests on their sensitivity, specificity.

**Results:**

We found that in all ELISA formats for FMD, antibody-trapping and competitive ELISAs have high specificity and RT-PCR (oligoprobing) ELISA has extra sensitivity. A panel of monoclonal antibodies to different sites or monoclonal antibody in combination of antiserum is the most suitable combination of antibodies in ELISA for FMD. Even though from its beginning, 3ABC is proven to be best performance in many studies, no single NSP can differentiate infected from vaccinated animals with complete confidence. Meanwhile, recombinant antigens and peptide derived from FMDV NPs, and NSPs have been developed for use as an alternative to the inactivated virus antigen for security.

**Conclusions:**

There is a need of target protein, which accurately determines the susceptible animal status based on the simple, fast and reliable routine laboratory test. A further alternative based on virus-like particle (VLP, also called empty capsids) in combination of high throughput antibody technique (Phage antibody library/antibody microarray) may be the powerful ELISA diagnostic reagents in future.

## Introduction

Foot and mouth disease(FMD) is a highly contagious and economically devastating disease of cloven-hoofed animals which hold a wide of the host spectrum such as cattle, pigs, sheep, goats, buffalo, deer, antelope and wild pigs and can severely constrain international trade of animals and animal products. FMD is caused by FMD virus (FMDV), a virus in the genus Aphthovirus within the family Picornaviridae [1]. The genome is over 8 kb in length and encode four structural proteins (SPs, VP1, VP2, VP3 and VP4)that form an icosahedrical capsid [2], and a total of ten mature non-structural proteins (NSPs)(L, 2A, 2B, 2C, 3A, 3B, 3C, 3D; or some complex, such as 3AB or 3ABC). Though the genome of FMDV is small, it has a high mutation rate and spontaneous. In FMDV, structural proteins are more variable than non-structural proteins. Mutations or deletions in structural proteins may help FMDV to evade an immune response produced by the host [3]. Furthermore, the variations are unequally distributed among the four structural proteins, particularly the VP1 protein, which shows the most frequently variability due to its significant roles in virus attachment, protective immunity, and serotype specificity. Antigenically, this virus exists as seven distinct serotypes (i.e., O, A, C, Asia 1 and SAT1-3) and multiple subtypes or antigenic variants within each serotype [4,5], which make the vaccine from one serotype does not confer protection against the other serotype. Consequently, vaccine strain requirements differ according to the type and subtypes of virus prevailing globally and the antigenic drift or antigenic shift of circulating virus or field isolates have to be survey on a large scale and matching vaccines have to be selected with care. Currently, vaccination remains the most effective countermeasure against FMDV, but, which complicated the problem of differentiate infected and vaccinated animals. Confront with parallel infection and vaccination, an accurate assessment to susceptible animal in a long range is urgent for determining the following control measures but also difficult due to lack of effective investigation approach. These limitations make the search for stable and safe test become an active area of research. In this review, the ELISA methodology and its utilization in the identification, detection and quantification of viral particle or viral antigens or specific antibodies are discussed. The newly reagent and skills, which show great promise but is still in the early stages of development was described as well.

### 1 ELISA for FMDV diagnosis/typing

Typical Clinical signs of FMD are characterised by a vesicular condition of the feet, buccal mucosa, rhinarium and the mammary glands of the females. Therefore, FMD cannot be differentiated clinically from other vesicular diseases, such as vesicular stomatitis, swine vesicular disease and vesicular exanthema. As a result, laboratory diagnosis of suspected FMD cases became a matter of significance.

Although methods based on virus isolation or the demonstration of FMD viral antigen or nucleic acid in samples of tissue or fluid or culture products is sufficient for a positive diagnosis, in general, the ELISA [6,7] using type-specific serological reagents is the preferred procedure for the detection of FMD viral antigen and identification of viral serotype in the early stages of research. Owing to it is more specific, sensitive and efficient, and it is not impacted by pro- or anti-complement factors [8] the ELISA has access to better development and even replaced complement fixation (CF) in most laboratories in the early research phase of FMD. Contrast to CF and virus isolation, almost the equivalent, even the higher of sensitivity was achieved in ELISA [9-11]. Different ELISA formats, particularly belonging to indirect ELISA, involved in blocking- or competition- or sandwich-based assays have been playing an increasing important role in the identification of FMDV serotype. The most common practice in the early period of the examination of FMD is that serotype-specific polyclonal antibodies (PAbs) or antiserum [7] to 146S (intact FMD virion) antigen of single or each of the seven serotypes of FMDV from rabbit and guinea-pig are used as trapping antibody and detecting antibody to develop a double-antibody sandwich ELISA (DAS-ELISA). In addition, the utilization of pre-coated microplate and freeze-dried reagents greatly extended those assays' applications field and shelf time [12]. However, there is experimental evidence that the similar level of neutralizing antibody against the first FMDV was stimulated by the heterotypic 146S particles and homotypic 146S particles. This displayed a similarity in the antigenic structure among the different serotypes and might sharply reduce the sensitivity of ELISA, which antibodies to 146S were adopted in. Practically, 12S particles, produced by mild acid treatment disruption of the 146S particle or by heating at 56°C, appear to be as active as 146S in inducing neutralizing antibody in guinea-pig inoculated with 146S particles [13-16], though there is a low neutralizing activity. Considering as a part of the 146S antigenic site, the 12S particles become a fresh substitute antigen for intact virus in serum preparation [11]. Further, an examination based on 12S particles was exploited as well [10]. However, a little deficiency existed in the abovementioned procedure is that the samples need to be treated to obtain subunits prior test. Antisera is important for many purposes, but they have some proportion of false-positive reactions or unexpected cross-reactivity owing to complex components. Moreover, the drawback related to limited production and heterogeneity lead to be difficult to standardization and hinder a large-scale application of antisera. As an alternative to guinea-pig or rabbit antisera, appropriate monoclonal antibodies (MAbs) secreted by specific hybridoma cells are considered the most promising of diagnostic reagents in terms of homogeneity and specificity and itself and peroxidase-conjugated can be used as capture antibody and detecting antibody, respectively.

A serotype-specific Mab-based antigen detection ELISA in use at the Istituto Zooprofilatico Sperimentale, Brescia, Italy [17], relies on a mixture of at least 3 different Mabs against each of the serotypes O, A and C to detect FMD virus in clinical samples. And complex-trapping-blocking [18] ELISAs using combinations of serotype-specific Mabs have been developed as well. Furthermore, numerous assays were built on the base of combination of PAbs and MAbs or two-MAbs system [19]. But it is worth pointing out that, in most cases, polyclonal antibodies are a better choice as coating antigen in former system and pairs of monoclonal antibodies subjecting to different antigen sites is necessary for the latter. In order to further enhance sensitivity, an approach of trace detection known as RT-PCR (oligoprobing) ELISA [20,21] in both solid and/or aqueous phase hybridization formats emerged and illustrated a bright prospect in mild subclinical infection or persistently infected carriers of lacking specific symptoms. Due to the highly contagious nature of FMD, the laboratory diagnosis related to live virus must be done in a laboratory that corresponds with the OIE requirements for Containment Group 4 pathogens. For the purpose of reducing the disease security risk involved compared with the use of live virus, inactivated antigens or trypsin-treated virus or proteins isolated by chemical or enzymic treatment of intact FMDV or recombinant proteins, particularly, the expressed VP1 protein [22] was proven to be a more accurate, safe target to examine FMDV and minimize the occurrence of false-positive results.

Currently, there is a little FMDV antigen detection test that employs the use of serotype-independent cross-reactive reagents. To aid improved diagnosis, Muller et al. [23] developed an assay system of serotype-independent FMDV antigen detection of all seven serotypes of FMDV using newly developed chickens antiserum targeting a highly conserved region located within the structural protein 1AB (accordingly named 1AB') [24] and a recently identified cross-serotype reactive monoclonal antibody recognized the N terminal of 1B [25].

### 2 ELISA for antigenic profile and epitopes analysis of FMDV

Owing the characteristics of quasispecies, natural isolate FMD viruses derived from different outbreaks showed much antigenic difference, which generally results in vaccine lacking effective protection against those FMD epidemic strains and even causing FMD outbreak. It is, therefore, an essential part of the epidemiological survey to assess regularly the antigenic characteristics of field isolates. Continuous monitoring of the antigenic relationship of field isolates in relation to the reference vaccine strain will provide an up to date knowledge about the efficacy of the vaccine virus in use and also has access to select the most suitable vaccine strains in case of emergency vaccination. Antigenic profiling by ELISA using panels of well-characterised MAbs [26] is suitable approaches for selecting representative virus isolates for vaccine matching. ELISA also has been used as a rapid method for assessing the relationship of vaccine virus strains with field virus strains collected [27] or those from outbreaks and subsequently, the liquid phase blocking (LPB) ELISA has been in use as an alternative to serum neutralization test (SNT) in determining the protective antibody response [28,29] and assessing the antigenic relationship of field viruses [30,31]. The result obtained in ELISA test was more appropriate towards incorporation of O1/Manisa into the vaccination program [31]. And the use of multiple Mabs has the advantage that it is less likely that a field virus will fail to possess at least one of the epitopes recognised by the antibodies. Antibodies that neutralize viral infectivity provide an important mechanism of protection against FMD. Thus, it is important to define epitopes, especially those ones that elicit the protective immune response and their variation or conservation among variants, subtypes and serotypes. In addition, the analysis of epitopes profile may provide significant reference for studying in aspects of molecular recognition, molecular immunology, particularly in the field of the selection of appropriate candidate vaccine strains. Traditional methods for defining linear antigenic epitopes, especially those neutralizing antigenic sites in more detail, include cloning and sequencing escape mutants [32], fragmentation of proteins either by chemical cleavage or by enzymatic digestion [33], peptide scanned technology based on peptide array or synthesizing a large number of peptides or a set of overlapping peptides corresponding to the known amino acid sequence of a protein [34,35]. Though some antigenic sites in FMD viruses of serotypes O, A and C and other Picornaviruses have identified mainly by abovementioned methods, ELISA- based approach identifying antigenic sites was still developed quickly. In 1985, McCullough et al. [36] firstly reported the usage of the liquid phase ELISA (LP-ELISA) in the FMDV epitope identification. Following, ELISA using single or an overlapping set of peptides has been used to map epitopes on VP1, VP2, VP3, VP4 [37-41]. Recently, another approach to map FMDV-NSP infection-related B-cell epitopes and T-cell epitopes by analyzing overlapping peptides which were used in ELISAs as synthetic peptides [32,42-44] was described.

Since monoclonal antibodies define a specific region, a large number of viruses can be analyzed against a panel of Mab in a single test [45]. Such studies [46-50] provide a rapid measure of the epitope profiles of viruses, because non-reactivity of a particular Mab is indicative of a minor antigenic difference between strains [51]. Nevertheless, a competition ELISA-based approach has been used successfully to define the epitopes of FMDV [47,52,53]. For confirmatory purposes, refinements to this approach such as the use of Fab fragments of antibodies or profiling using site-specific mutant viruses should be considered. Furthermore, to estimate the relative proportion of anti-FMDV antibodies with different antigenic site specificities presenting in the antiserum from cattle, swine and sheep, conventionally immunized with O1 serotype vaccine, Aggarwal et al. [54] has used a capture competition ELISA in his work. As described for a non-serotype specific antigen detection ELISA, the identification of an epitope shared between all of the seven serotypes of FMD virus could be the basis of a non-serotype specific competition ELISA able to detect antibody to any strain of FMD virus. Due to their highly conserved nature, epitopes on the NSP's of the virus are the most likely candidates for such a site.

### 3 ELISA for differentiating infected from vaccinated animals (DIVA)

Control of FMD mainly encompasses vaccination and slaughter policy, particularly for the FMD-free countries, and slaughter policy is considered a fundamental measure. However, some restrictions from economic situation, social culture, geographical and natural environment limit this policy to be applied in a large-scale range in the endemic areas. Consequently, vaccination or the combination policy of vaccination and slaughter remain the most effective countermeasure against FMDV. Though different type of vaccine comprising subunit vaccine, peptide vaccine, DNA vaccine are developed, FMD inactivated vaccine still play a key role in control campaigns and eradication of FMD [55] in the majority FMD epidemic countries and territories because of perfect protection potency. However, another new problem arises from inactivated vaccine of FMD is that it is difficult to distinguish vaccinated from infected animals. And then, A range of ELISA techniques are currently being evaluated with the intention of producing ELISAs for routine diagnostic use which are capable of detecting antibody to FMD virus NSP's. These methods were described as following:

**3D **Protein 3D, also known as the virus infection associated antigen (VIAA) since antibodies to this antigen would be detected in serum from recovery animals [56], is the core subunit of the virus-encoded RNA-dependent RNA polymerase [57] and responsible for proteolytic cleavage and viral replication [58].

The antigenicity of 3D protein is highly conserved among all serotypes [59], holding out the possibility of a single serological test capable of detecting infection with any of the seven serotypes of the virus. 3D polymerase is the first NSP to be used to distinguish FMDV infected from vaccinated animals. The traditional VIAA is a semi-purified antigen prepared from the virus grown in tissue culture. When it is used in ELISA, there are problems of inadequate reproducibility appearing to be an inherent characteristic of the semi-purified nature of the VIAA preparations (World Reference Laboratory, unpublished findings). In order to address these problems, recombinant VIAA antigen has considerable attractions. When used in a simple indirect ELISA, recombinant 3D can differentiate infected from naive cattle [60]. The sensitivity of the test is only slightly lower than the conventional liquid phase blocking ELISA of Hamblin et al. [61] and the specificity is approximately 95%. Moreover, the 3D antibody tests can be used to monitor viral activity in large cattle populations and for certification of FMDV free animals for import and/or export testing [59]. Whereas the following research showed that repeatedly vaccinated animals can develop antibodies to 3D, which demonstrate 3D is insufficient to differentiate infection from vaccination.

**3B **Protein 3B, also known as VPg, is the viral genome-bound protein. It exists in three nonidentical copies and covalently bound to the 5' end of the genome and serves as a primer for virus genome replication [62]. Besides, the VPg copy number also has a substantial association with the host range and virulence [63]. An epitope-blocking ELISA (EB-ELISA) exploring combination of MAb to 3B core repeat motif (QKPLK) and purified recombinant 3AB protein was developed by Oem et al. [64] to evaluate FMD-free herds and vaccinated cattle, pigs, goats, and sheep in 2007.

**3ABC **The polypeptide 3ABC, playing an important role during the different stages of viral replication [65-67], is perceived by the most researchers as the most appropriate antigen to distinguish infection from vaccination because of its high immunogenicity and relatively low concentration in FMDV-infected cell lysates. Numerous different mode of 3ABC-ELISAs based on the E.coli expression system were developed to discriminate naïve, infected, vaccinated pig/cattle/sheep and detect silent infections/subclinical cases, or survey field samples in FMD-vaccinated or infected populations [68-71]. In order to imitate native protein as far as possible, the NSPs expressed in baculovirus expression systems [58,72,73] was searched. In 2003, Kweon et al. [74] established Mab linked indirect-trapping ELISA using the baculovirus expressed 3ABC (mainly amino acid 1417-1835) and MAb against 3A. The equal sensitivity and specificity were acquired in comparing with commercial kits (baculovirus expressed 3ABC I-ELISA from USDA and Mab (3A) linked E. coli expressed 3ABC I-ELISA from IZSLE) during retrospective sero-surveillance. Then, Sørensen et al. [75] modified the method of Kweon et al. and removed non-specific reactions in sera of cattle and sheep by filtration and inactivation. Due to the antigen capture strategy can sharply simplify the purification step of recombinant protein, Clavijo et al. [76] established a biotinylated 3ABC competitive ELISA(cELISA), which demonstrated no differences between species (cattle, sheep, pigs) and virus serotypes. Other indirect and competitive ELISAs detecting antibodies to 3ABC have been shown to have equivalent diagnostic performance characteristic [77,78].

However, the NSPs expressed in E. coli and baculovirus expression systems may sometimes create problems in the interpretation of the results on account of non-specific reactions. Furthermore, the number of epitopes found on such a long recombinant protein may interfere with antibodies to other Picornaviruses [43,79,80]. Moreover, chemically synthesized synthetic peptides of NSPs were used for DIVA [43,80-82]. Subsequently, Foord et al. [83] provided a C-ELISA format on the basis of complete bacterial expression systems in 2007. The combination of recombinant antibody single chain variable fragments (scFv) from phage display libraries with E.coli-derived recombinant 3ABC reflected the best performance in detecting sera from cattle, sheep and pigs representing naïve, FMDV-vaccinated or FMDV-infected animals. The biggest advantage of this test is safe, economical and without the need for infectious virus, the use of laboratory animals or the costly maintenance of viable hybridoma cell lines. The results indicated that scFv displayed the potential to replace polyclonal or monoclonal antibodies in such assays.

**Other NSPs or NPs **ELISAs based on other NSPs and NPs or assays using infection-specific epitopes of NSPs to remove cross reactivity are being explored [43,80,81]. Moreover, chimeric proteins that infection related B-cell epitopes of FMDV NSPs, which self-assembled into chimeric tymovirus-like particles(TVLPs), was selected as a candidate antigen for use in I-ELISA for DIVA of different species from the field [84]. The function of the nonstructural proteins 2B is not very clear, although it has hydrophobic domains [85,86]. However, a 2B peptide ELISA, described by Inoue et al. [42], using a chemically synthesized 2B peptide (RSTPEDLERAEKQ) as antigen showed more competitive strength than other NSP tests in the detection of the early period of infection because of the characteristic of earlier induction (as early as 1-2 weeks after infection) and longer persistence of the antibody to 2B. Then, Ko et al. [87] reported rP13C ELISA in 2009, which explored the recombinant protein (rP13C) expressed in insect cells as a diagnostic antigen. The higher endpoint titers than LPB-ELISA and virus neutralization test (VNT) was represented in the measure of sera from goats challenged with FMDV post-vaccination.

### 4 ELISA for quantification of FMDV

Usually, the effectiveness or immunogenicity of the vaccine depends to a large extent on the content of 146S and the stability of these particles after virus inactivation procedures and formulation into vaccines. In the past, the most common method for the quantification of the FMD whole virus particle is either CsCl, or linear sucrose density centrifugation procedure [88], both of which are labor intensive, time-consuming, require expensive equipment and cannot assess whether the virus has been affected by proteolytic enzymes. In recent years, as a rapid, effective serological method, ELISA, which not only sharply simplified the detection process, but also greatly increased sensitivity and specialty, is emerging crucial and novel application in terms of evaluation the amount of intact virus in vaccine. Moreover, ELISA can simultaneously quantify 146S in many samples and monitor whole virus particle in each step of vaccine manufacture as well as being used to test the effects of medium modification and different culture models. Since whole particles and subunits share most of the common epitopes, polyclonal sera against purified 146S particles cross react with subunits. In order to overcome above cross-reactivity, one strategy for the specific detection of 146S based on pairs and single MAb, which bound only to 146S and not to the subunit particle was demonstrated by Van Manaan et al. [89] and Crowther et al. [90] in the presence of virus subunits (12S), respectively. Then, during in-process controls, Alonso et al. [91] quantified antigen mass adopting the panels of MAb to FMD types O, A and C in an indirect sandwich ELISA (IS-ELISA). In 2008, Yang et al. [92] further optimized the method of Van Manaan et al. by employing polyclonal antibodies as the capture antibody and promoted the quantification of 146S of FMDV serotype O and A to nanogram level. In 2010, Capozzo et al. [93] firstly developed an in-process control filtration-assisted chemiluminometric immunoassay to quantify FMDV non-capsid proteins in vaccine-antigen batches. A detection limit, 2 ng for purified NCP and 4 ng for vaccine-antigen batches spiked with NCP, was gained in this method.

## Conclusion

FMD, which is an acute, contagious disease of cloven-hoofed animals, has caused huge economic losses since the finding and is still prevalent in many parts of the world. Though different approaches are used, the ELISA assay has been actively studied in terms of examination, variation, and diagnosis research of FMD. A preliminary study of ELISA was aimed at typing FMDV different serotype, and subsequent was used to identify antigen epitopes and evaluate the relationship or the variant degree between reference virus and isolated strains or circulating strains. Currently, ELISA was designed to exploit their potential in differentiating infected from vaccinated animals or carriers and quantitation virions during different stages of vaccine production, particularly in manufacture of pre-production, in-process and final products test. Although theoretically the detection of antibody to NSP's must indicate infection rather than vaccination, but in practice, antibody against NSPs also might be provoked by trace amounts of NSP's existed in commercial vaccines and multiple vaccinations. Therefore, currently no test has been fully validated. Whereas, a popular viewpoint from international NSP test validation at Brescia is that the usage of multi-NSP test will acquire the most suitable combination of tests and increase the efficiency of detection [77,94]. Furthermore, innovation and exploit related to ELISA formats was developed as well. Starting with indirect ELISA, the liquid-phase blocking ELISA (LPBE)[61,28] and the solid-phase competitive ELISA(SPCE) [17,95-97] were advocated. Other trap and competitive ELISAs detecting animal antibody from different species have shown better diagnostic performance. These ELISAs either use purified antigens absorbed directly to microplates or use polyclonal or monoclonal antibodies to trap specific antigens from semi-purified preparations [69,73,98,99].

Regardless of the target (antigens or serum), as the core reagent of ELISA, antibody, particularly a large number of monoclonal antibody against FMD virus different regions of SP and NSP was prepared [100-107]. Accordingly, MAb based ELISAs make advances and improvement, particularly those used in antigen mapping. In order to eliminate cross-reactivity as soon as possible and increase the specificity, an approach combining antiserum from rabbit or guinea-pig and monoclonal antibody was exploited and perform well in the examination of FMD. In addition, antibody produced from non-animals, such as single-chain antibody, may show a bright future as a promising ELISA reagent.

The risk of virus release is the primary consideration for most researchers in the choice of virus antigen. To address this limitation, recombinant proteins and peptide derived from FMDV NPs and NSPs have been developed for alternative to whole-virus and inactivated virus antigen. However, the narrow antigenic epitopes harboring in such antigens have generally been insufficient to either afford solid protection of natural hosts or potent test. But empty capsids maintain continuous and discontinuous B-cell epitopes and T-cell epitopes presenting in authentic virions [108-114] and may induce protective responses similar to those elicited by inactivated virus. At present, empty capsids of FMDV of different serotypes have been obtained by using either Escherichia coli or baculovirus as an expression system [115,116], especially a recent report of the versatility of the baculovirus expression system [117] for the production of P12A3C precursor of FMDV. Therefore, as a further alternative to peptide or simple recombinant protein, empty capsids of FMDV will open new hotspot in the field of both ELISA reagent and vaccine antigen.

## Competing interests

The authors declare that they have no competing interests.

## Authors' contributions

LNM contributed to the original draft of the manuscript, and approved the final version. JZ and HTC contributed to conception and design of the manuscript, and involved in revising the manuscript. JHZ and YZD helped to provide information and suggestion. YSL is the corresponding author. All authors read and approved the final manuscript.
